# Acute effects of blue and red light exposure on cognitive performance, exercise capacity, perceived effort, and dynamic balance: A randomized crossover study

**DOI:** 10.1016/j.jesf.2025.08.003

**Published:** 2025-08-13

**Authors:** Ukbe Sirayder, Merdan Orunoglu, Oguzhan Yilmaz

**Affiliations:** a Department of Physiotherapy and Rehabilitation, Faculty of Health Sciences, Nuh Naci Yazgan University, Kayseri, Turkey; b Kayseri State Hospital, Neurosurgery Clinic, Kayseri, Turkey; c Department of Therapy and Rehabilitation, Vocational School of Higher Education, Nuh Naci Yazgan University, Kayseri, Turkey

**Keywords:** Blue light, Reaction time, Cross-over studies, Fatigue, Postural balance

## Abstract

**Background:**

This study investigated the acute effects of narrow-band blue (460 nm) and red (630 nm) light exposure on cognitive performance, exercise capacity, perceived fatigue, and dynamic balance in healthy young males. To our knowledge, this is the first randomized crossover study to systematically assess the combined impact of pre-exercise light exposure on both cognitive-motor integration and aerobic performance within an exercise context.

**Methods:**

Fifty physically active young males participated in a randomized crossover design. Participants were exposed to either blue or red light, followed immediately by assessments of simple reaction time (SRT), Incremental Shuttle Walk Test (ISWT) distance, heart rate, perceived exertion, and Y-Balance Test performance.

**Results:**

Blue light exposure led to a significant and large improvement in SRT (Δ = −53.33 ms; p < 0.001, η^2^_p_ = 0.270) and enhanced dynamic balance. Red light exposure produced greater increases in ISWT distance (Δ = +36.98 m; p = 0.004, η^2^_p_ = 0.453) and significant reductions in perceived fatigue and dyspnea. A moderate positive correlation was observed between SRT improvement and ISWT distance under blue light (β = 0.1869, p = 0.008).

**Conclusion:**

This study demonstrates that short-term, wavelength-specific light exposure may optimize both cognitive and physiological readiness prior to exercise. These findings provide novel evidence supporting the integration of individualized light-based strategies in athletic preparation protocols.

## Highlights

• This is the first study combining light exposure with cognitive-exercise tasks.
• Cognitive and motor benefits were wavelength-specific and outcome-dependent.
• Blue light significantly improved reaction time and dynamic balance performance.
• Red light exposure increased aerobic capacity and reduced perceived exertion.
• Blue light-induced alertness correlated with walking performance under fatigue.

## 1 Introduction

Exercise performance is a multidimensional construct influenced not only by training status and physical capacity, but also by environmental and neurophysiological factors. In this context, the effects of light exposure—particularly at specific wavelengths—on human performance have garnered increasing attention in both sports science and neuroscience. This growing interest is grounded in the concept of photobiomodulation, which posits that light within certain spectral bands can modulate cellular metabolism, neurotransmission, and psychophysiological states.^1^

Beyond wavelength alone, the effects of light are modulated by factors such as illuminance, spectral composition, exposure timing, and individual characteristics (e.g., pupil diameter, chronotype, age).^2^^,^^3^ Brown (2020) emphasized the role of melanopic illuminance—measuring stimulation of intrinsically photosensitive retinal ganglion cells (ipRGCs)—in predicting non-visual effects like melatonin suppression and circadian shifts.^2^ Similarly, Nie et al. (2024) showed that light with higher illuminance and specific spectral content (e.g., red-enriched white light) enhances alertness and working memory, with minimal melatonin suppression.^3^ These findings highlight that light-induced outcomes depend on a complex interplay of optical and biological variables.

Current literature on red light (approximately 630 nm) predominantly consists of in vitro or tissue-targeted experimental studies. These studies often show that red light enhances mitochondrial adenosine triphosphate production by stimulating cytochrome *c* oxidase, thereby promoting muscular endurance and recovery.^4^^,^^5^ However, most of these findings originate from direct tissue exposure models, with limited translational relevance to ocular applications in humans.

Human studies examining ocular red light exposure in exercise contexts are extremely scarce. One of the few exceptions is Zhao et al. (2012), who found that nighttime red light exposure improved performance on a 12-min run test. This effect was attributed to enhanced melatonin secretion and improved sleep quality, suggesting an indirect, sleep-mediated performance mechanism.^6^

In contrast, blue light (approximately 460 nm) exhibits shorter wavelengths and higher photon energy, primarily acting via retinal melanopsin receptors. This interaction enhances alertness, attention, and cognitive speed. Even brief exposure has been shown to improve reaction times and cognitive accuracy in laboratory tasks.^7^ However, such studies have largely been limited to static laboratory environments and have failed to assess the integration of these cognitive enhancements into exercise-related outcomes.^6^, ^7^, ^8^

Most prior research has examined red and blue light independently, often focusing exclusively on either physiological or cognitive outcomes—rarely both. Furthermore, crossover-design studies that evaluate cognitive and physical performance in tandem under differing light conditions are lacking. Existing research on blue light tends to focus on vigilance or isolated response time measures, without extending to ecologically valid, exercise-integrated settings.

To date, no study has systematically compared the acute effects of blue and red light exposure on both physical and cognitive performance within a crossover design that integrates real-world exercise tasks in healthy adults. Despite the strong theoretical rationale supporting wavelength-specific neural and metabolic effects, integrative experimental studies assessing both cognitive and physical outcomes within a single protocol remain remarkably limited.

Therefore, the present study aimed to compare the acute effects of narrow-band red and blue light exposure—administered immediately prior to exercise—on cognitive performance, exercise capacity, perceived effort, and dynamic balance in healthy young adults. The findings aim to offer new insights into wavelength-specific optimization of pre-exercise protocols in sports and performance-based clinical applications. In line with this aim, it was hypothesized that blue light exposure would lead to greater acute improvements in cognitive performance and dynamic balance, while red light exposure would result in greater enhancements in exercise capacity and reductions in perceived effort.

## 2 Methods

### 2.1 Participants

An a priori power analysis was conducted using G∗Power 3.1 to determine the minimum required sample size for the present 2 × 2 repeated-measures crossover design. Based on the findings of Tonetti et al. (2018), who reported a moderate-to-large effect size (Cohen's d ≈ 0.75) for the acute impact of blue light exposure on reaction time, the corresponding partial eta squared (η^2^_p_) was estimated at approximately 0.12 ^7^. With a 95 % confidence level (α = 0.01) and 80 % statistical power (1–β = 0.80), the analysis indicated that a minimum of 36 participants would be required to detect significant interaction effects between light condition and time. To accommodate potential dropouts and increase statistical robustness, 50 participants were recruited. This sample size provides sufficient power to detect reliable within-subject differences and evaluate the wavelength-specific effects of light exposure on cognitive and physical performance outcomes in an exercise-integrated setting.

This study was conducted between January 15, 2024, and February 1, 2025, at the MCORE Performance and Sports Facilities affiliated with the Rectorate of Erciyes University. A total of 50 healthy volunteers aged between 20 and 30 years, all of whom engaged in regular physical activity, were included in the study. All participants were of Turkish ethnicity. Eligible participants reported a history of engaging in exercise at least three times per week over the previous six months and were non-smokers. Only individuals with a normal body mass index (18.5–24.9 kg/m^2^) were included.

Participants were required to have normal uncorrected vision; those using eyeglasses or contact lenses were excluded due to the potential influence of optical correction on light perception. Individuals with a known history of photosensitivity-related neurological conditions (e.g., migraine, epilepsy), sleep disorders, musculoskeletal disorders, or recent surgical interventions were excluded. Additionally, participants undergoing pharmacological treatment or with a documented history of systemic diseases (including cardiovascular, endocrine, or neurological conditions) were not eligible.

**Fig. 1:**
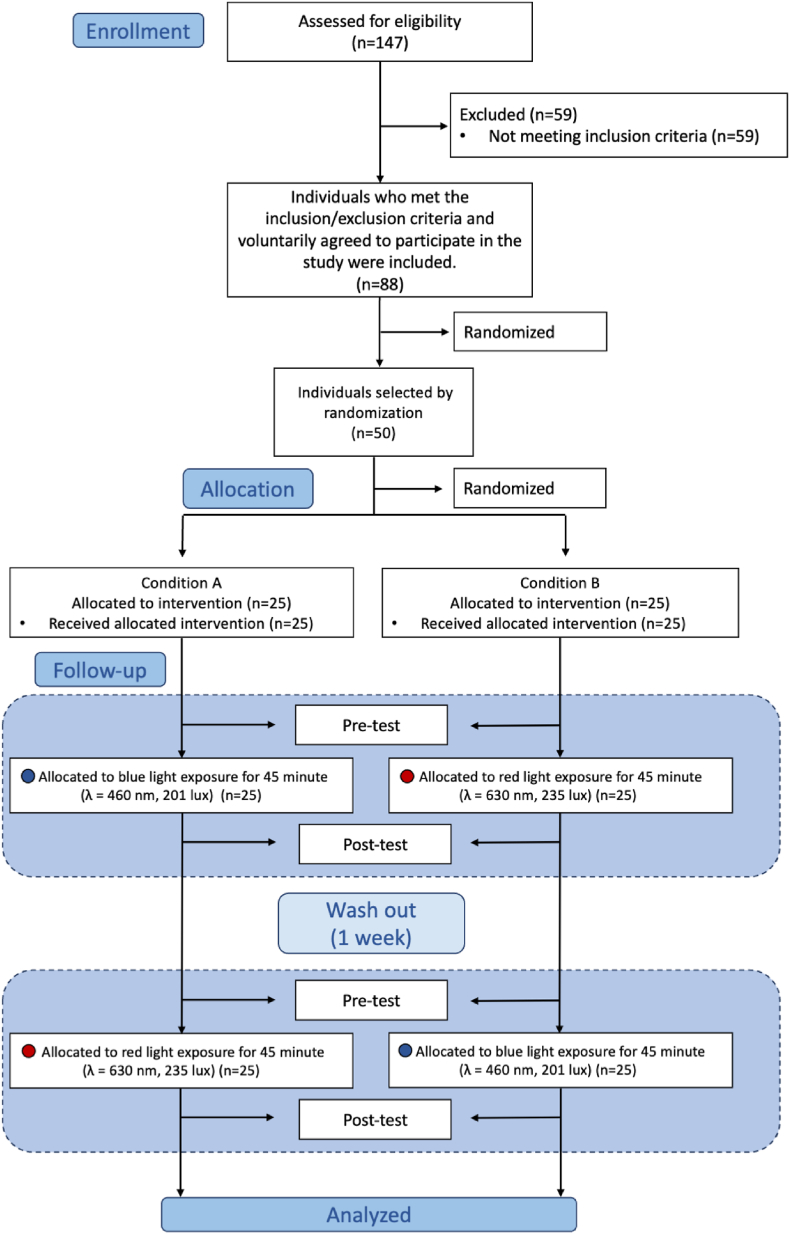
Flow chart.

Initially, 147 individuals who regularly attended the MCORE Center for physical activity were identified. Among them, 88 individuals met the predefined inclusion and exclusion criteria. Subsequently, 50 participants were randomly selected using a computer-based randomization algorithm (www.random.org). These 50 individuals were then assigned to initial exposure conditions (red or blue light) using block randomization to ensure balance across sessions in the crossover design. This multi-step sampling approach was designed to minimize selection bias and ensure methodological transparency in participant allocation (Fig. 1).

All participants were thoroughly informed about the study's objectives, procedures, and potential risks prior to testing and provided written informed consent. They were instructed to refrain from intense physical activity, caffeine and alcohol consumption, and to ensure adequate sleep during the 24 h preceding the testing sessions.

### 2.2 Experimental design

**Fig. 2:**
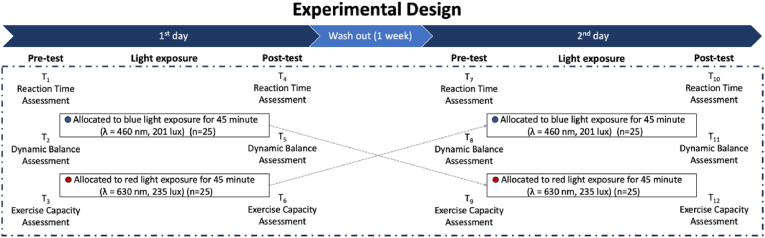
Schematic representation of the experimental design.

This study employed a randomized, comparative, cross-over design in which each participant was exposed to two different light conditions in separate sessions: red light exposure at a wavelength of 630 nm and blue light exposure at a wavelength of 460 nm (Fig. 2). Half of the participants (n = 25) underwent red light exposure during the first session, while the remaining participants (n = 25) received blue light exposure. In the second session, each participant was exposed to the alternate light condition, ensuring that all individuals were tested under both conditions. The sequence of light exposure was randomized using computer-assisted block randomization. In both sessions, participants were exposed to the assigned light condition for 45 min in a controlled laboratory environment prior to testing. Identical performance assessments were conducted before (pre-test) and after (post-test) the light exposure. The pre-test measurements reflected the participants’ baseline physiological and cognitive states, while the post-test assessments aimed to capture the acute effects of light exposure. A minimum 1-week wash-out period was implemented between sessions to minimize carry-over effects and ensure physiological recovery. This cross-over, within-subjects design allowed each participant to serve as their own control, thereby reducing inter-individual variability and enhancing statistical power.

As part of the experimental timeline and procedural controls, all test sessions were conducted between 10:00 a.m. and 12:00 p.m. under standardized laboratory conditions to minimize the influence of circadian rhythms and behavioral variables. Participants were instructed to obtain at least 7 h of sleep the night before each test session, refrain from consuming caffeinated beverages and alcohol for at least 12 h prior to testing, and avoid strenuous physical activity for 24 h before the session.

To avoid potential confounding effects due to fatigue accumulation, the sequence of testing within each session was carefully structured. The simple reaction time test was administered first, followed by the Y-Balance Test (YBT) to assess dynamic postural control, and finally, the Incremental Shuttle Walk Test (ISWT) was conducted. This specific order was chosen because the ISWT is a near-maximal test that can lead to residual fatigue, which might otherwise affect the outcomes of more cognitively or neuromuscularly sensitive assessments if performed earlier in the protocol (Fig. 2).

Participants were not blinded to the intervention due to the overt visual difference between red and blue light. Similarly, outcome assessors were not blinded, as the assessments involved standardized, objective measurements (e.g., computerized reaction time, validated physical performance tests) that were not prone to subjective interpretation. Nevertheless, the lack of assessor blinding remains a potential source of bias and should be considered when interpreting the results. Although subjective ratings (e.g., perceived exertion) were self-reported, assessors did not influence these responses; however, the absence of blinding could still have introduced an expectancy effect.

This study was conducted and reported in accordance with the CONSORT 2010 guidelines for randomized crossover trials.

### 2.3 Light exposure protocol

The light exposure protocol used in this study was implemented based on previously established procedures reported in the literature.^9^ Participants were exposed to light for 45 min in a controlled laboratory setting immediately prior to the testing session.

Light was delivered using the Waldmann® Biolux HCL system (Herbert Waldmann GmbH & Co. KG, Germany), a programmable device capable of emitting narrow-band light in both blue (λ = 460 nm) and red (λ = 630 nm) spectral ranges. For the blue light condition, the device was configured to emit 460 nm light at 201 lux, while for the red light condition, the system delivered 630 nm light at 235 lux, as specified by the manufacturer and in accordance with existing photobiological research protocols.

**Fig. 3:**
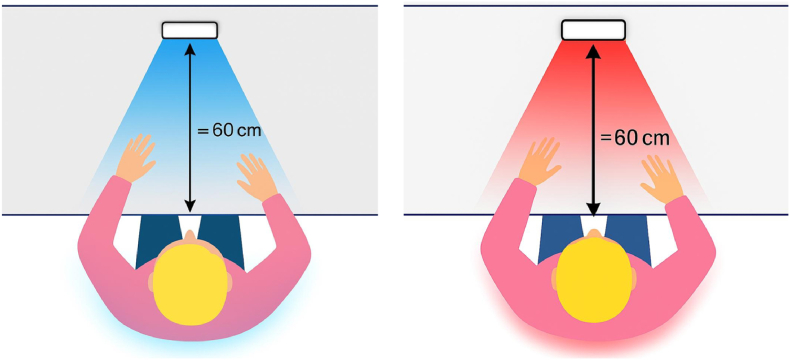
Experimental setup of blue and red light exposure conditions.

The light exposure was administered from a distance of 60 cm at a 90-degree angle directly in front of the participants’ eyes, ensuring optimal stimulation of retinal melanopsin receptors (Fig. 3). The LED panel used in the experiment provided uniform and stable illumination across the visual field. During the exposure, they were instructed to sit quietly without engaging in cognitive tasks or using electronic devices, to maintain consistent exposure conditions. Ambient lighting in the room was minimized, and the test environment was isolated from external light sources to ensure consistent luminance across sessions.

This standardized protocol aimed to investigate the acute neuromodulatory effects of narrow-band light exposure on both physical and cognitive performance when administered immediately before exercise.

### 2.4 Measurements

#### 2.4.1 Reaction time assessment

Reaction time was assessed following light exposure to examine short-term changes in cognitive alertness and visual processing speed. In this study, only a simple reaction time test was administered.

Participants were instructed to press the spacebar key to a standard keyboard with their dominant hand as quickly as possible upon the appearance of a green circular visual stimulus (approximately 10 cm in circumference) at the center of a digital screen. The assessment was conducted via the Cognitive Fun platform (http://cognitivefun.net/test/1), operating at a sampling rate of 500 Hz.^10^, ^11^, ^12^ All tests were performed in a controlled laboratory environment with participants seated approximately 55 cm from the screen. Each subject completed three consecutive trials, with a 30-s rest interval between each trial to minimize anticipatory responses. Prior to data collection, a brief familiarization session was provided to ensure consistency in task understanding and execution.^13^

The average reaction time across the three trials, recorded in milliseconds (ms), was used for statistical analysis. This protocol, validated in previous cognitive neuroscience research, offers a reliable and sensitive measure of psychomotor response speed in response to external visual cues following acute light stimulation.

#### 2.4.2 Exercise capacity assessment

The ISWT was administered to evaluate participants’ maximal aerobic capacity in a standardized and progressive manner. The test was performed over a 10-m course marked by two cones, requiring participants to walk back and forth in synchrony with audio signals emitted from a pre-recorded CD. The test began at a walking speed of 0.5 m/s, with each subsequent level increasing in speed by 0.17 m/s at regular intervals. Participants continued the test until they were unable to maintain the required pace due to fatigue or voluntarily chose to stop.^14^ Throughout the procedure, physiological and perceptual responses were monitored. Heart rate, blood pressure, oxygen saturation (Cosmed Spiropalm 6MWT, Milan, Italy), respiratory rate, perceived dyspnea, and overall fatigue (assessed using the Modified Borg Scale) were recorded both before and immediately after the ISWT.^15^ The total distance covered was documented as the primary performance outcome.

#### 2.4.3 Dynamic balance assessment

Dynamic balance was assessed using the YBT, a validated tool designed to evaluate lower extremity stability, proprioceptive acuity, and neuromuscular control. Prior to testing, all participants were informed about the test procedures and allowed to complete a brief familiarization session. During the test, participants stood on one leg while reaching with the opposite leg in three directions: anterior, posteromedial, and posterolateral. The goal was to reach as far as possible without compromising balance. Any trial was deemed invalid and repeated if the participant lost balance, shifted the stance foot, or moved the hands above the hip line. Each reach direction was tested three times per limb, and the best distance was recorded for analysis. Reach distances were normalized to leg length using the formula:

%reachdistance=(reachdistance/leglength)×100

This approach reduced the confounding effects of individual anthropometric variation on performance outcomes. The composite score, representing an overall balance performance indicator, was calculated by averaging the maximum reach distances in all three directions (anterior, posteromedial, and posterolateral) and then normalizing the sum to leg length:

Compositescore(%)=[(ANT+PM+PL)/(3×leglength)]×100

ANT: Anterior, PM: Posteromedial, PL: Posterolateral.

This score provides a single metric reflecting dynamic balance across multiple planes of movement.^16^

### 2.5 Statistical analysis

All statistical analyses were conducted using IBM SPSS Statistics (Version 27.0, IBM Corp., Armonk, NY, USA) and GraphPad Prism (Version 10.0, GraphPad Software, San Diego, CA, USA). Descriptive data are presented as mean ± standard deviation (SD). The normality of distribution for continuous variables was assessed using the Shapiro–Wilk test, supported by visual inspection of histograms and Q–Q plots. To evaluate the effects of light exposure (red vs. blue) and time (pre-test vs. post-test), two-way repeated-measures ANOVA was performed, with “Time” as the within-subject factor and “Condition” as the repeated factor. The Condition × Time interaction was examined to assess differential responses to light across time. Effect sizes were reported using partial eta squared (η^2^_p_), interpreted as small (≥0.01), medium (≥0.06), and large (≥0.14). All primary analyses, including the repeated-measures ANOVA and effect size reporting, were pre-specified in the study protocol. Additionally, Pearson correlation analyses were used to examine associations between post-test outcomes within each light condition. For any significant associations, simple linear regression analysis was conducted to explore predictive relationships. All statistical assumptions—including normality, sphericity, and homogeneity of variance—were checked and met. The threshold for statistical significance was set at α = 0.05 for all analyses.

## 3 Results

A total of 50 healthy male volunteers participated in the study. The mean age of the participants was 26.41 ± 4.55 years, the average height was 174.78 ± 8.24 cm, and the mean body mass index (BMI) was 20.11 ± 2.33 kg/m^2^. All participants completed both light exposure sessions without reporting any adverse effects. Participant compliance with pre-test instructions and procedural protocols was high, indicating good tolerability and methodological feasibility. All participants completed both test sessions, and no dropouts occurred. This high retention rate is likely attributable to the brief, low-burden nature of the protocol, the participants’ habitual physical activity, and effective engagement strategies.

**Fig. 4:**
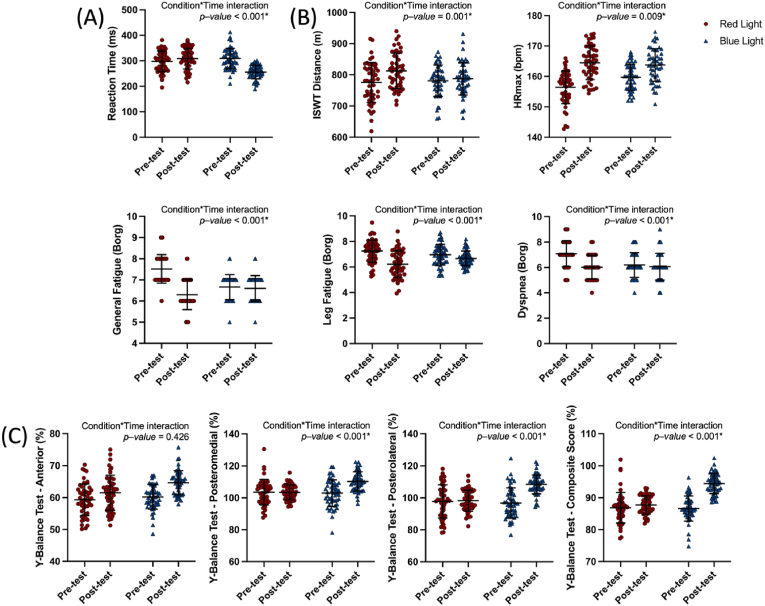
Effects of light exposure (red vs. blue light) on physiological, perceptual, balance, and cognitive performance outcomes before and after the intervention. ∗p < 0.05. (A) Simple reaction time (ms) results showing cognitive response speed. (B) Changes in heart rate max (HRmax), Incremental Shuttle Walk Test (ISWT) distance, general fatigue, leg fatigue, and dyspnea (Borg scale) scores before and after the intervention under red and blue light conditions. (C) Y-Balance Test performance across anterior, posteromedial, posterolateral directions, and composite scores. Data are presented as individual values with mean ± standard deviation.

Repeated-measures ANOVA revealed significant main effects of time, as well as significant interactions between light condition (red vs. blue) and time (pre-test vs. post-test) across all outcome variables (Table 1, Fig. 4).

**Table 1:** Effects of light exposure on physiological, perceptual, balance, and reaction time outcomes.

Variables		Pre-test	Post-test	Within-Condition Change Scores^ϕ^	Main Effect (Time)	Between-Condition Difference in Change Scores^ϕ^	Main Effect (Condition)	Condition∗Time interaction	
		Mean ± SD	Mean ± SD		*p*^*1*^ – value		*p*^*2*^ – value	F/*p*^*3*^ – value	η^2^_p_
***Reaction Time***									
Simple Reaction Times (ms)	**Red Light**	297.49 ± 39.70	308.57 ± 40.87	11.08 (−4.98 – 27.15)	<0.001^a^	−64.42 (−85.62–−43.20)	<0.001^a^	36.39/<0.001^a^	0.270
	**Blue Light**	308.84 ± 40.17	255.51 ± 26.45	−53.33 (−67.59–−39.06)					
***Exercise Test***									
ISWT Distance (m)	**Red Light**	775.44 ± 64.09	812.43 ± 56.61	36.98 (11.94–62.02)	0.004^a^	30.16 (−14.50 – 28.14)	0.068	11.55/0.001^a^	0.453
	**Blue Light**	780.84 ± 50.57	787.66 ± 52.33	6.82 (−14.50 – 28.14)					
HR_max_ (bpm)	**Red Light**	156.40 ± 5.39	164.48 ± 5.51	8.08 (5.72–10.44)	<0.001^a^	6.06 (4.56–7.57)	0.076	7.035/0.009^a^	0.067
	**Blue Light**	159.74 ± 4.30	163.79 ± 5.36	3.93 (1.94–5.91)					
General Fatigue (Borg)	**Red Light**	7.52 ± 0.68	6.30 ± 0.71	−1.22 (−1.36–−1.08)	<0.001^a^	−1.16 (−1.32–−0.99)	0.039^a^	197.17/<0.001^a^	0.801
	**Blue Light**	6.60 ± 0.61	6.66 ± 0.59	0.06 (−0.15–−0.03)					
Leg Fatigue (Borg)	**Red Light**	7.26 ± 0.79	6.33 ± 1.04	−1.35 (−1.43–−1.26)	<0.001^a^	−1.32 (−1.14–−0.76)	0.023^a^	256.31/<0.001^a^	0.843
	**Blue Light**	6.89 ± 0.84	6.73 ± 0.61	−0.03 (−0.19 – 0.01)					
Dyspnea (Borg)	**Red Light**	7.08 ± 1.02	6.02 ± 0.93	−1.06 (−1.22–−0.90)	<0.001^a^	−0.92 (−1.13–−0.70)	0.031^a^	74.916/<0.001^a^	0.605
	**Blue Light**	6.20 ± 0.96	6.06 ± 1.05	−0.14 (−0.25–−0.03)					
***Y – Balance Test***									
Anterior (%)	**Red Light**	60.5 ± 5.3	61.7 ± 5.4	2.18 (−0.04 – 4.41)	0.154	−2.29 (−4.88 – 0.30)	0.002^a^	0.63/0.426	0.006
	**Blue Light**	60.1 ± 4.4	64.3 ± 4.2	4.47 (2.82–6.13)					
Posteromedial (%)	**Red Light**	102.4 ± 7.3	103.2 ± 6.0	0.11 (−2.74 – 2.53)	<0.001^a^	−7.38 (−11.24–−3.52)	<0.001^a^	14.30/<0.001^a^	0.061
	**Blue Light**	103.1 ± 7.7	109.1 ± 6.1	7.27 (4.38–10.16)					
Posterolateral (%)	**Red Light**	98.2 ± 9.7	100.1 ± 7.2	0.51 (−3.09 – 4.11)	<0.001^a^	11.13 (6.46–15.80)	<0.001^a^	20.89/<0.001^a^	0.176
	**Blue Light**	97.3 ± 8.9	108.2 ± 6.3	11.64 (8.33–14.95)					
Composite Score (%)	**Red Light**	86.85 ± 4.78	87.71 ± 2.89	0.85 (−0.74 – 2.45)	<0.001^a^	6.94 (5.08–8.80)	<0.001^a^	42.65/<0.001^a^	0.125
	**Blue Light**	86.62 ± 3.93	94.41 ± 3.14	7.79 (6.32–9.27)					

All outcomes were analyzed using repeated-measures ANOVA with within-subject factor “Time” (Pre vs. Post) and between-subject factor “Light” (Red vs. Blue). Partial eta squared (η^2^_p_) was reported as a measure of effect size. A significance threshold of *p* < 0.05 was applied for all comparisons.

a p < 0.05. ^ϕ^(95 % confidence interval), SD: Standard Deviation, *p*^*1*^: Within group comparison (Time), *p*^*2*^: Group comparison (Group), *p*^*3*^: Between group change score comparison.

A significant Condition × Time interaction was also observed for simple reaction time (F(1,49) = 36.39, p < 0.001, η^2^_p_ = 0.270; large effect) (Table 1, Fig. 4-A). Reaction times improved substantially under blue light exposure (Δ = −53.33 ms, 95 % CI [−67.59 to −39.06], p < 0.001), whereas a non-significant increase was noted under red light exposure (Δ = +11.08 ms, 95 % CI [−4.98 to 27.15]).

A significant Condition × Time interaction was observed for ISWT distance (F(1,49) = 11.55, p = 0.001, η^2^_p_ = 0.453; large effect) (Table 1, Fig. 4-B). While both light exposures improved ISWT performance, the increase was more pronounced under red light (Δ = +36.98 m, 95 % CI [11.94–62.02], p = 0.004) compared to blue light (Δ = +6.82 m, 95 % CI [−14.50–28.14]). For HRmax, a significant Condition × Time interaction was found (F(1,49) = 7.035, p = 0.009, η^2^_p_ = 0.067; medium effect). HRmax values increased significantly from pre- to post-test in both groups. Under red light, HRmax increased by +8.08 bpm (95 % CI [5.72–10.44], p < 0.001), while under blue light, the increase was +3.93 bpm (95 % CI [1.94–5.91], p < 0.001). However, the between-condition difference in HRmax change scores (+6.06 bpm, 95 % CI [4.56–7.57]) did not reach statistical significance (p = 0.076). Significant Condition × Time interactions were also found for perceived general fatigue (F(1,49) = 197.17, p < 0.001, η^2^_p_ = 0.801; very large effect), leg fatigue (F(1,49) = 256.31, p < 0.001, η^2^_p_ = 0.843; very large effect), and dyspnea (F(1,49) = 74.92, p < 0.001, η^2^_p_ = 0.605; large effect). In all three parameters, significant reductions were greater under red light exposure, with substantial within-condition improvements and significant between-condition differences (all p < 0.05).

Regarding dynamic balance outcomes (YBT), significant Condition × Time interactions were found for posteromedial reach (F(1,49) = 14.30, p < 0.001, η^2^_p_ = 0.061; medium effect), posterolateral reach (F(1,49) = 20.89, p < 0.001, η^2^_p_ = 0.176; large effect), and composite score (F(1,49) = 42.65, p < 0.001, η^2^_p_ = 0.125; large effect) (Table 1, Fig. 4-C). Improvements were significantly greater under blue light, particularly for posterolateral reach (Δ = +11.64 %) and composite balance score (Δ = +7.79 %). No significant interaction was found for anterior reach (F(1,49) = 0.63, p = 0.426, η^2^_p_ = 0.006; negligible effect), though within-condition improvements were still observed under blue light (Δ = +4.47 %).

**Fig. 5:**
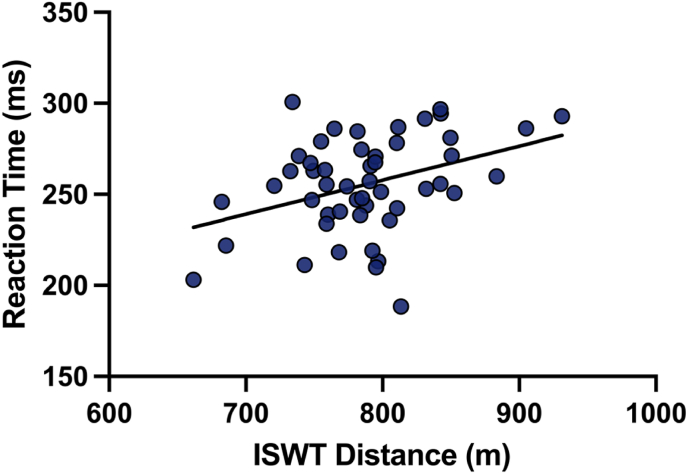
Correlation between ISWT distance and post-intervention simple reaction time under blue light condition. The linear regression line is shown with a shaded 95 % confidence band. The relationship was statistically significant (r = 0.37, p = 0.008, R^2^ = 0.137), indicating that increased walking distance was associated with faster reaction times.

Regression analysis revealed a moderate but statistically significant positive relationship between ISWT distance and simple reaction time under blue light (β = 0.1869, 95 % CI [0.0506–0.3232], p = 0.008), suggesting that greater improvements in aerobic performance were associated with faster cognitive processing speed. The intercept was estimated at 108.3 ms (95 % CI [0.73–215.9]), and the standard deviation of residuals (Sy.x) was 24.83 ms. No significant correlations were found between simple reaction time and other measured variables (HRmax, fatigue scores, or balance parameters) under either light condition (p > 0.05) (Fig. 5).

## 4 Discussion

In this study, the acute effects of red and blue light exposure on maximal exercise capacity, cardiovascular responses, perceived fatigue, dynamic balance, and cognitive performance outcomes were evaluated using a multidimensional approach. The findings indicate that different wavelengths of light, when applied during the pre-exercise period, can induce specific alterations in both physiological and neurocognitive parameters. Specifically, red light exposure was associated with modest improvements in aerobic performance and reductions in perceived exertion, whereas blue light exposure led to greater enhancements in cognitive responsiveness and postural balance. This suggests a wavelength-dependent divergence in how photic stimuli modulate readiness for exercise across physical and cognitive domains.

Simple reaction time, as a fundamental index of cognitive arousal and sensorimotor coordination,^17^ improved significantly following blue light exposure (Δ = −53.33 ms), indicating enhanced cognitive alertness. This effect is consistent with previous research suggesting that blue light activates melanopsin-containing retinal ganglion cells, stimulating brain regions involved in attention such as the suprachiasmatic nucleus, locus coeruleus, and prefrontal cortex.^8^^,^^18^ Since testing occurred in the morning when melatonin levels are low, these effects likely reflect direct neural stimulation rather than circadian shifts.

Tonetti et al. demonstrated that even a brief, 1-min exposure to blue light resulted in significant improvements in semantic priming and attentional network components (alerting, orienting).^7^ Similarly, studies reported that short-term exposure to blue light enhances attention, reaction speed, and overall cognitive arousal.^19^^,^^20^ However, the majority of these findings were obtained from passive cognitive tasks conducted in controlled laboratory settings.

The observed enhancement in reaction time may be partly explained by the neurophysiological effects of short-wavelength blue light. Light in the 460–480 nm range activates melanopsin-containing ipRGCs, which project to the suprachiasmatic nucleus, the locus coeruleus (LC), and other subcortical structures involved in arousal and attention regulation. Through this melanopsin–LC–prefrontal cortex axis, blue light may enhance cortical norepinephrine transmission, thereby modulating attention, vigilance, and executive control functions.^21^ However, recent findings suggest that the cognitive impact of blue light is context-dependent and may be influenced by color-related expectations or learned associations. For example, Yang et al. (2023) reported that blue-light exposure impaired exogenous attention shifting, potentially due to culturally acquired negative connotations associated with the “blue light hazard”.^22^ Despite these contextual complexities, the improved SRT performance observed in our study may reflect a dominant neuromodulatory effect of ipRGC-mediated stimulation under conditions optimized for alertness and cognitive readiness.

In this respect, this study is among the first to examine the integrated effects of pre-exercise blue light exposure on both motor and cognitive domains. The results suggest that blue light may enhance cognitive alertness not only in laboratory tasks but also in environments involving physical activity, potentially contributing to improved athletic performance.

These improvements, observed under morning light exposure, are unlikely to be mediated by melatonin suppression—which is minimal during early-day hours—but rather reflect direct ipRGC stimulation pathways. This aligns with emerging neurophysiological models suggesting that blue light enhances prefrontal attentional readiness via LC–norepinephrine activation, independently of circadian phase modulation.^23^ Thus, blue light stimulation may serve as a non-pharmacological cognitive enhancer in settings demanding rapid perceptual-motor coordination, such as pre-competition routines or cognitive rehabilitation sessions.

In this study, significant improvements in ISWT distance were observed following both red and blue light exposure; however, the increase was statistically more pronounced under the red light condition (Δ = +36.98 m vs. Δ = +6.82 m). This finding is noteworthy, as such a substantial enhancement in ISWT performance following acute red light exposure has not been previously reported in the literature.

Preliminary experimental evidence suggests that transcranial application of red light may induce cerebrovascular responses. For instance, in the study conducted by Rungta et al., it was demonstrated that direct application of red light through the skull could lead to localized increases in blood flow within superficial brain regions. However, it is important to note that these findings pertain specifically to direct cerebral surface illumination and do not reflect non-invasive, ocular-based red light exposure.^24^ In our study, the intervention was designed to induce systemic and indirect photic stimulation, primarily targeting central neural structures such as the suprachiasmatic nucleus via retinal melanopsin pathways. Therefore, attributing the observed improvements in exercise performance and perceived exertion directly to enhanced cerebral blood flow remains speculative within the scope of our current data. In summary, although the hypothesis that red light exposure may modulate cerebral circulation finds some indirect support in the literature, the underlying mechanisms were not directly assessed in our study. As such, definitive conclusions regarding this pathway cannot be drawn and warrant further investigation.

Recent data imply that red light exposure may enhance sympathetic, rather than parasympathetic, activity. Ross et al. reported a reduction in heart rate variability following red light stimulation, indicating increased sympathetic tone.^25^ In this context, the improvement in ISWT distance observed in our study may be linked to acute sympathetic activation, potentially enhancing cardiovascular alertness and motivation during exercise. However, as autonomic parameters were not directly measured, this interpretation remains speculative and warrants further investigation. Moreover, emerging evidence from Zhao et al. indicated that 14 days of whole-body red-light irradiation enhanced endurance performance in elite female basketball players, possibly mediated by improvements in sleep quality and melatonin levels.^6^ Although the aerobic test used (Cooper 12-min run) differs from the ISWT utilized in our study, both assess maximal cardiovascular endurance. It is conceivable that enhanced recovery and neuroendocrine regulation induced by red-light exposure may have facilitated better exercise tolerance in both contexts. However, given that our protocol employed a single-session, retinal-focused photic stimulation rather than chronic whole-body exposure, direct comparisons must be approached cautiously. Further studies are warranted to delineate the chronic versus acute effects of red-light interventions on aerobic capacity.

Although previous literature frequently reports that red light enhances mitochondrial function, these effects are generally localized to the tissue directly exposed.^26^ In our study, light was administered through the eyes, aiming for systemic rather than localized effects. Therefore, the observed improvements in ISWT performance are more likely attributable to mechanisms such as central nervous system activation, improved sleep-related recovery, and reduced perceived exertion,^6^ rather than enhanced mitochondrial energy production. Nonetheless, recent studies^3^^,^^5^^,^^9^^,^^23^ have also reported acute effects of red light exposure in humans, such as transient changes in autonomic activity, arousal modulation, and perceptual responses—suggesting that ocular red light may exert short-term physiological influences even in the absence of direct mitochondrial stimulation.

Nevertheless, the ergogenic effects observed in this study likely stem from non-mitochondrial mechanisms, given the short exposure duration and the indirect (ocular) delivery method. A study suggests that red light may acutely influence autonomic nervous system balance by enhancing sympathetic drive and motivational arousal—especially relevant during submaximal to near-maximal effort tasks.^25^ Although speculative, this pathway may explain the observed reductions in subjective fatigue and dyspnea. These effects warrant further exploration in real-world athletic contexts where short, targeted light exposures could enhance readiness without requiring direct tissue illumination.

Additionally, both red and blue light exposure led to significant increases in HRmax. This result suggests that participants experienced higher levels of physiological stress post-exposure, potentially indicating reduced exercise efficiency. Although the increase in HRmax was more pronounced under the red light condition, it is not straightforward to interpret this finding as being in alignment or contrast with prior studies favoring blue light. Regardless, the clinical relevance of the observed differences remains limited despite statistical significance.

Consistent with our findings on exercise capacity, subjective indicators such as general fatigue, leg fatigue, and dyspnea were significantly reduced following red light exposure, whereas no substantial changes were noted under blue light conditions. Given that perceived fatigue and respiratory discomfort are critical markers of internal workload during exercise,^27^ these reductions suggest a meaningful alleviation of subjective exercise burden. Although the underlying mechanisms remain speculative, it is conceivable that red light exposure may influence central nervous system pathways associated with motivational and exertional regulation. Additionally, prior research has shown that red light exposure under resting conditions may promote parasympathetic activity, as indicated by increases in high-frequency heart rate variability.^28^ However, during exercise, the physiological demands may favor a shift toward sympathetic activation, aligning with our observed increases in HRmax and enhanced exercise capacity.^25^ Therefore, it is plausible that the observed improvements in ISWT performance were partially mediated by central nervous system effects and a dynamic autonomic adjustment, collectively contributing to both reduced subjective fatigue and superior exercise performance following red light exposure.

In the current study, significant improvements were observed in dynamic balance parameters following blue light exposure, particularly in posterolateral reach and composite scores, both demonstrating large effect sizes (η^2^_p_ = 0.176 and 0.125, respectively). These findings suggest that short-term blue light stimulation may acutely enhance complex motor control processes involved in postural stability.

The enhancement in balance performance under blue light can plausibly be attributed to its well-established effects on cognitive functions such as attention, alertness, and executive control. Through activation of melanopsin-containing retinal ganglion cells and subsequent stimulation of the suprachiasmatic nucleus, blue light exposure has been shown to promote wakefulness and improve cognitive processing speed.^8^ Tonetti et al. demonstrated that even a brief, 1-min exposure to blue light significantly reduced reaction times and modulated attentional networks.^7^ Given the integration between cognitive processes and proprioceptive feedback mechanisms in postural control, these cognitive enhancements may translate into superior balance performance.

Although no significant Condition × Time interaction was observed for anterior reach, within-condition improvements were still noted under blue light. Importantly, the substantial gains in posterolateral reach (Δ = +11.64 %) and composite balance scores (Δ = +7.79 %) highlight that blue light exposure may particularly benefit tasks requiring dynamic postural adjustments, lateral stability, and complex sensorimotor coordination. These results suggest that acute blue light stimulation could be strategically utilized as a preparatory intervention to enhance balance-related performance in athletic or rehabilitation contexts. This neuromotor facilitation may be particularly beneficial in sports or rehabilitation environments requiring rapid adaptation to balance challenges (e.g., return-to-play assessments, dual-task gait training, fall prevention programs). Additionally, because the observed improvements were achieved using only short-term ocular exposure, blue light may offer a time-efficient priming strategy with minimal logistical burden—provided that lighting conditions are safely adapted to individual sensitivity and environmental constraints.

In contrast, red light exposure did not yield significant improvements in dynamic balance measures. This is consistent with the hypothesis that red light predominantly influences physiological and metabolic processes rather than directly enhancing neurocognitive or motor control domains.^25^ Combined with our findings regarding exercise capacity and perceived exertion, these balance results emphasize that the spectral properties of photic stimuli may differentially affect various neuromotor outcomes.

In the present study, a significant and large effect of blue light exposure was observed on simple reaction time, with participants demonstrating a substantial improvement (Δ = −53.33 ms) compared to baseline. No significant change was detected under red light exposure (Δ = +11.08 ms). These findings align with previous evidence suggesting that blue light stimulation enhances cognitive responsiveness by activating melanopsin-containing retinal pathways and modulating central nervous system arousal.^7^^,^^8^ However, unlike prior studies that primarily assessed isolated cognitive tasks in static conditions, our investigation explored the integrated effects of photic stimulation on both cognitive and physical performance parameters.

Our regression analysis revealed a statistically significant and moderately positive relationship between ISWT distance and simple reaction time improvements under blue light exposure (β = 0.1869, p = 0.008). This result suggests that acute enhancements in cognitive alertness may positively influence functional aerobic capacity, highlighting a cognitive-motor interplay that could be leveraged in athletic or clinical settings. Specifically, improvements in reaction speed may facilitate more efficient decision-making, neuromotor activation, and pacing strategies during dynamic physical tasks such as incremental shuttle walking.

Notably, this relationship was exclusive to the blue light condition and was not observed with other variables (e.g., HRmax, fatigue scores, or balance outcomes) or under red light exposure. These findings suggest a degree of specificity whereby blue light-induced cognitive enhancements may translate into superior exercise performance, potentially via improved cognitive-motor integration. This observation complements our balance findings, where blue light exposure also resulted in superior postural control improvements, further supporting the notion that cognitive arousal induced by photic stimulation can exert meaningful effects on motor outcomes.

Nevertheless, given that the identified correlation was moderate in strength and confined to a specific outcome pair, caution is warranted when interpreting these results. Future research employing comprehensive cognitive and neuromotor assessments is needed to fully elucidate the mechanisms underpinning the observed associations and to determine whether such effects are generalizable across different populations, task complexities, and light exposure protocols.

To our knowledge, this is the first study to integrate acute light exposure with concurrent cognitive and exercise performance tasks within a crossover framework. These findings support a potential model in which blue light exposure enhances prefrontal cortex activity through LC-norepinephrine signaling, thereby improving top-down attentional control.^29^ In the context of physical exertion, such enhanced cognitive readiness may facilitate more efficient pacing, motor planning, and task engagement—ultimately leading to improved aerobic performance. This light–cognition–exercise triad warrants further investigation through combined neuroimaging and performance-based paradigms to better characterize how cortical networks dynamically respond to pre-exercise photic stimulation.

Although the present study employed narrow-band light exposure to isolate wavelength-specific effects, it remains essential to evaluate the translational relevance and safety of these findings in real-world settings. Most everyday environments utilize polychromatic light sources with broad spectral compositions. Notably, light enriched in short wavelengths (i.e., blue light) has been associated with potential risks such as circadian disruption, melatonin suppression, and phototoxicity.^30^ Therefore, the spectral content and intensity of light should be carefully considered when adapting experimental protocols for practical implementation. Moreover, while the alertness-enhancing effects of blue light are commonly attributed to melatonin suppression, this mechanism is primarily relevant during evening and nighttime hours.^31^ Since the present study was conducted in the morning and early afternoon, the observed cognitive improvements are more likely attributable to direct activation of arousal-related brain regions via ipRGCs. Similarly, although red light is widely known for its long-term photobiomodulatory effects at the mitochondrial level, recent evidence suggests that even brief ocular exposure can exert acute autonomic and neuromodulatory effects.^32^

### 4.1 Limitations

This study has several limitations that warrant consideration. First, the sample consisted exclusively of young, healthy male participants, which limits the generalizability of the findings to broader populations, including females, older adults, and clinical groups. Second, no direct neurophysiological or biochemical assessments (e.g., cerebral blood flow, autonomic activity, hormonal responses) were conducted, thereby restricting mechanistic interpretations. Third, the intervention involved a single acute light exposure session of fixed duration, which precludes conclusions regarding chronic effects or dose-response relationships. While the selected outcome measures provide valuable insights into cognitive, cardiovascular, and neuromotor domains, their applicability to high-intensity exercise or elite performance contexts remains uncertain. Despite employing a randomized crossover design with a one-week washout, residual or carryover effects may still have occurred. Future studies should consider incorporating objective physiological or biochemical washout markers to confirm the independence of crossover phases. Furthermore, repeated exposure to testing procedures may have resulted in learning or habituation effects, potentially influencing cognitive or balance-related outcomes. Another limitation is the absence of a control, sham, or placebo condition, which restricts the ability to distinguish the effects of light exposure from general practice-related improvements. Future research should address this by including appropriate control conditions. Moreover, chronotype, habitual sleep duration, and individual light sensitivity—factors known to influence cognitive arousal and circadian responses—were not formally assessed and should be accounted for in future research using validated chronobiological tools. Lastly, although the reaction time assessment tool used in this study (Cognitive Fun, http://cognitivefun.net/test/1) has been employed in previous experimental studies, no formal validity or test–retest reliability analyses are available in the literature. As a result, the psychometric properties of this tool remain uncertain and should be taken into account when interpreting the findings.

### 4.2 Recommendations for future research

Building on these initial findings, several avenues warrant further investigation. First, future studies should explore the long-term effects of repeated light exposure to distinguish between acute and chronic adaptations. Expanding research to include diverse populations—such as females, older adults, clinical groups, and elite physically active populations—would enhance the external validity of the current results.

Future studies should incorporate neuroimaging (e.g., fNIRS, EEG) and autonomic markers (e.g., HRV, hormones) to clarify the mechanisms involved. Additionally, systematic manipulation of light exposure parameters—such as wavelength, intensity, and duration—could help clarify dose-response relationships and optimize intervention protocols.

Importantly, further studies should investigate the interaction between light exposure and different types of physical activity, including high-intensity interval training, endurance exercise, resistance training, and balance-specific tasks. Such research would support the development of targeted, sport-specific, and individualized light-based preparation strategies aimed at enhancing both cognitive and physical performance.

From an applied perspective, the light system used in this study (Biolux HCL) offers the advantages of controlled and programmable wavelength exposure, while also being relatively low-cost and accessible. These features may facilitate the widespread implementation of such protocols in practical settings such as gyms, clinics, or workplaces. Evaluating the feasibility of alternatives like LED panels, screen filters, or wearable devices may help identify more cost-effective options. These adaptable approaches may enhance the translational value of light-based interventions and promote their broader application across athletic, occupational, and clinical domains.

## 5 Conclusion

This study is among the first to investigate the acute effects of red and blue light exposure on cognitive performance, aerobic capacity, perceived fatigue, and dynamic balance in healthy young males. Importantly, it is the first to examine the influence of pre-exercise photic stimulation on both cognitive and physical performance within an integrated framework. The findings indicate that red light may be associated with modest improvements in exercise capacity and reductions in perceived exertion, whereas blue light may enhance cognitive responsiveness and postural control. Although the effects were statistically significant, they warrant cautious interpretation due to the exploratory design and the absence of direct physiological measures.

These preliminary results suggest that wavelength-specific light exposure may serve as a modifiable environmental factor that can acutely influence performance-related outcomes. This approach may hold relevance not only for athletic preparation but also for other high-performance domains such as emergency response, surgical procedures, military operations, or competitive esports.

## Author statement

All authors contributed significantly to the conception, design, data collection, analysis, and/or interpretation of the study. The manuscript is original, has not been published previously, and is not under consideration elsewhere. All authors have read and approved the revised version of the manuscript submitted in response to the reviewers’ comments. The authors collectively agree with the content and confirm the accuracy and integrity of the final version. The authors declare no conflicts of interest. Ethical approval and informed consent were obtained in accordance with institutional and international guidelines. Corresponding author: Dr. Ukbe SIRAYDER.

## Availability of data and materials

The datasets generated and analyzed during the current study are available from the corresponding author on reasonable request.

## Ethics approval and consent to participate

The study protocol was approved by the Scientific Research and Publication Ethics Committee of Nuh Naci Yazgan University (Approval No: 2024/001–04, dated January 4, 2024). Written informed consent was obtained from all participants prior to enrollment.

## Consent for publication

Not applicable.

## Availability of data and materials

The datasets generated and analyzed during the current study are available from the corresponding author on reasonable request.

## Funding

This research did not receive any specific grant from funding agencies in the public, commercial, or not-for-profit sectors.

## Competing of interests

The authors declare that they have no known competing financial interests or personal relationships that could have appeared to influence the work reported in this paper.
